# Adaptive capacity at the northern front: sockeye salmon behaviourally thermoregulate during novel exposure to warm temperatures

**DOI:** 10.1093/conphys/cow039

**Published:** 2016-10-04

**Authors:** Jonathan B. Armstrong, Eric J. Ward, Daniel E. Schindler, Peter J. Lisi

**Affiliations:** 1 Department of Fisheries and Wildlife, Oregon State University, 104 Nash Hall, 2820 SW Campus Way, Corvallis, OR 97331,USA; 2 Northwest Fisheries Science Center, National Oceanic and Atmospheric Administration, 2725 Montlake Boulevard East, Seattle, WA 98112,USA; 3 School of Aquatic and Fishery Sciences, University of Washington, Box 355020, Seattle, WA 98105,USA; 4 Center for Limnology, University of Wisconsin, 680 North Park Street, Madison, WI 53706,USA

**Keywords:** Climate change, latitude, refuge, thermal heterogeneity

## Abstract

As climate change increases maximal water temperatures, behavioural thermoregulation may be crucial for the persistence of coldwater fishes, such as salmonids. Although myriad studies have documented behavioural thermoregulation in southern populations of salmonids, few if any have explored this phenomenon in northern populations, which are less likely to have an evolutionary history of heat stress, yet are predicted to experience substantial warming. Here, we treated a rare heat wave as a natural experiment to test whether wild sockeye salmon (*Oncorhynchus nerka*) at the northern extent of their primary range (60° latitude) can thermoregulate in response to abnormally high thermal conditions. We tagged adult sockeye salmon with temperature loggers as they staged in a lake epilimnion prior to spawning in small cold streams (*n* = 40 recovered loggers). As lake surface temperatures warmed to physiologically suboptimal levels (15–20°C), sockeye salmon thermoregulated by moving to tributary plumes or the lake metalimnion. A regression of fish body temperature against lake surface temperature indicated that fish moved to cooler waters when the epilimnion temperature exceeded ~12°C. A bioenergetics model suggested that the observed behaviour reduced daily metabolic costs by as much as ~50% during the warmest conditions (18–20°C). These results provide rare evidence of cool-seeking thermoregulation at the poleward extent of a species range, emphasizing the potential ubiquity of maximal temperature constraints and the functional significance of thermal heterogeneity for buffering poikilotherms from climate change.

## Abstract (precis)

Salmon at high latitudes face rapid climate change and a future with new physiological challenges. A heat wave in 2013 provided a glimpse of warmer temperatures to come, and northern sockeye salmon rose to the challenge, moving to coldwater refuges and avoiding heat stress.

## Introduction

Temperature is a heterogeneously distributed ecological resource with fundamental significance to animal physiology (Magnuson *et al*., 1979; Huey, 1991). A key challenge for many animals is to maintain a physiologically optimal body temperature as environmental temperatures vary over time. One way that animals cope with thermal variation in time is to exploit variation in space, moving to refuge habitats that remain physiologically suitable as other locations become too warm or cold (Huey, 1991). This behavioural thermoregulation occurs in species ranging from larval fish that migrate vertically in lakes (Wurtsbaugh and Neverman, 1988) to large terrestrial mammals that rest in the shade (Sargeant *et al*., 1994). Thermoregulation is a salient issue for conservation because it has the potential to buffer species from climate change (Sears *et al*., 2011; Sunday *et al*., 2014; Woods *et al*., 2015), yet its capacity may be diminished by human development that fragments or homogenizes habitats (e.g. agriculture, hydropower; Fausch *et al*., 2002; Schindler *et al*., 2015).

Thermoregulation is thought to be most important in populations at the latitudinal extents of a species range, where environmental temperatures are most likely to exceed cold and warm thermal tolerances (i.e. CT_min_ and CT_max_, respectively; Sunday *et al*., 2014). In particular, cooling thermoregulation is anticipated to be a crucial coping mechanism for low-latitude populations experiencing warming associated with global climate change (Sunday *et al*., 2014). A key unknown is how high-latitude populations will respond to the rapid levels of warming that they are already beginning to experience with ongoing climate change. These populations have historically had high thermal safety margins such that annual maximal temperatures were unlikely to become physiologically suboptimal (i.e. exceed the *pejus* temperature or approach CT_max_). Therefore, these populations may not have used thermoregulation to maintain optimal body temperatures in suboptimally warm environments, but may need to under future warming. Although the threats of warming may be less severe for high-latitude populations compared with low-latitude populations, they are still relevant to conservation because they may mediate the abundance and productivity of commercially, culturally or ecologically significant species. For example, the extirpation of an already dwindling population at low latitudes could have less ecological and economic impacts than a productivity decline in an abundant high-latitude population. Furthermore, high-latitude populations may be more sensitive to warming than anticipated if they are locally adapted to their thermal regimes (Eliason *et al*., 2011).

In freshwater ecosystems, behavioural thermoregulation can be crucial for the persistence of fishes that are physiologically adapted to cold water (e.g. salmonids). A large body of work has documented various salmonids reducing body temperature by moving to habitat patches that remain relatively cool during periods of warm weather (Biro, 1998; Torgersen *et al*., 1999; Newell and Quinn, 2005; Breau *et al*., 2011; Dugdale *et al*., 2015) Although cool-seeking behavioural thermoregulation is well documented in salmonid populations at the core or southern extent of their distributions, there is very little known about how this behaviour varies latitudinally. Northern populations have been shown to exhibit warm-seeking thermoregulation in response to suboptimally cool temperatures (Armstrong and Schindler, 2013), but few studies have explored behavioural responses to heat stress (Westley *et al*., 2010). Although the basic attributes of thermotaxis may be conserved within species, successful thermoregulation may be rather complex, requiring individuals quickly to locate patches of refuge habitat spread widely across watersheds and to balance movement to cool water with competing needs of migrating, breeding or avoiding predators. Indeed, some salmonid populations fail to thermoregulate and experience suboptimally high temperatures even when coldwater refuges are present on the riverscape (Donaldson *et al*., 2009; Strange, 2012). Studying behavioural responses to heat stress in northern populations is challenging because stressful warm temperatures may be encountered rarely and they are difficult to induce experimentally. However, weather anomalies during summer provide natural experiments in which ephemerally warm conditions induce novel exposure to heat stress, simulating future conditions under climate change.

Here, we assess whether sockeye salmon (*Oncorhynchus nerka*) at the northern extent of their range (60° latitude) exhibit behavioural thermoregulation in response to atypical periods of potentially stressful temperatures. Sockeye salmon rear in lakes as juveniles, spend their adult lives in the Pacific Ocean, and then return to freshwater streams and lake shores to spawn (Quinn, 2005). Sockeye salmon cease feeding after returning to freshwater (Armstrong, 2010), so the costs of migration, maturation and spawning are fuelled by stored energy (Hendry and Berg, 1999). Prior to spawning, sockeye salmon aggregate along lake shores in close proximity to spawning habitats, such as tributary streams (Hodgson and Quinn, 2002). During this staging period at the end of their migration, individuals have already consumed much of their energy stores (Hendry and Berg, 1999) and may be particularly sensitive to the effects of temperature on metabolic costs (Brett, 1971), pathogenicity and immunocompetence (Lafferty, 2009; Bradford *et al*., 2010). Furthermore, these pre-spawning aggregations often distribute near the surface of the lake, where the epilimnion may be microstratified during calm weather and highly sensitive to changes in air temperature and incident solar radiation. Although northern salmon stocks are typically abundant and forecasted to experience milder heat stress (in absolute terms) than southern populations, the ability of northern populations to respond to rapid warming is relevant to conservation because the productivity of these salmon stocks strongly affects regional economies and ecosystems, and northern stocks may be locally adapted to relatively cool conditions (Eliason *et al*., 2011) that make them more sensitive to new extreme thermal events.

In 2013, watersheds at the northern extent of the sockeye salmon range experienced warm temperatures during the mid-summer period when adult salmon stage and spawn. To examine whether high-latitude populations exhibit behavioural thermoregulation in response to a rare episode of heat stress, we opportunistically tagged fish with temperature loggers and recorded the water temperatures they selected in a thermally heterogeneous lake. Our objectives were as follows: (i) to test for thermoregulation and characterize the range of temperatures that fish select; (ii) to quantify individual-level variation in thermoregulatory behaviour; and (iii) to evaluate the energetic implications of any observed thermoregulation (e.g. the effect on potential freshwater lifespan).

## Materials and methods

### Study system

This research was conducted in the Wood River watershed of Southwest Alaska, which consists of five major lakes that drain into Bristol Bay. Wood River sockeye salmon are one of nine major stocks contributing to the Bristol Bay fishery. In recent decades (1950–2008), the Bristol Bay fishery produced 68% of all sockeye salmon caught in the USA (Schindler *et al*., 2010). The ~1 million salmon that spawn in the Wood River every year provide a primary food source for several species of fish and wildlife and support subsistence harvest by local communities. The Wood River watershed is located near the northern extent of the geographical range of sockeye salmon. The primary distribution of sockeye salmon extends only one major drainage basin north of the Wood River, to the Kuskokwim River (~60–63° latitude; Groot and Margolis, 1991).

**Figure 1:**
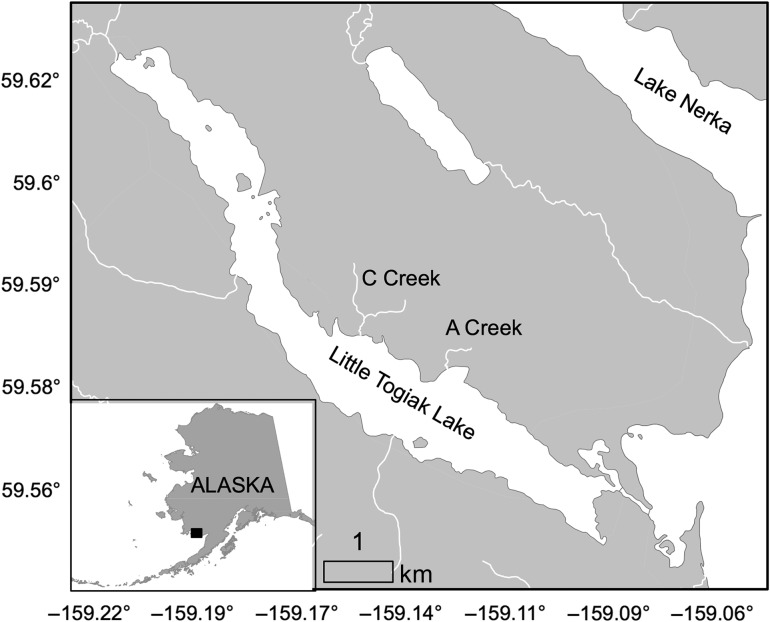
A map of the study system, Little Togiak Lake, AK, USA. Sockeye salmon were tagged with temperature loggers in the lake epilimnion near C Creek.

Sockeye salmon adults return to the Wood River system from the Bering Sea during a condensed period in early summer. Historically (1961–2011), 80% of the run has occurred between 29 June (±4.2 days) and 11 July (±2.6 days; Alaska Department of Fish and Game, unpublished data). After freshwater entry, sockeye salmon reside in the Wood River watershed's lakes or connecting rivers, where they mature and develop secondary sexual traits prior to spawning, which occurs from mid-July to early October depending on local habitat conditions (Lisi *et al*., 2013). During the period between freshwater entry and spawning (i.e. the staging period), sockeye salmon form conspicuous aggregations in the lake epilimnion, often near tributary inlets to lakes and along shorelines. In 2013, we monitored sockeye salmon staging in Little Togiak Lake (Fig. 1), which is thermally stratified and supports several genetically distinct populations of sockeye salmon, two of which spawn in small very cold streams (A and C Creeks; Peterson *et al*., 2014). Peak spawning in these cold streams occurs in mid-August.

### Monitoring temperatures used by sockeye salmon

Using a beach seine, we captured sockeye salmon as they aggregated along the lakeshore near the mouth of C Creek. As part of a concurrent, long-term monitoring project, fish were anaesthetized with clove oil, measured for body length, depth and maturity, and then tagged with Petersen discs. We sealed calibrated iButton temperature loggers (Dallas Semiconductor, Dallas, TX, USA; ±0.5°C accuracy) between two 4 cm × 4 cm pieces of Gorilla Tape (Gorilla Glue, Cincinnati, OH, USA) and then skewered the tape to place it between the Petersen disc and the fish (the tape allowed some moisture in, so we do not recommend this method for longer-term studies). We tagged 95 fish on 27 July. The iButtons recorded temperature at 20 min intervals and were recovered from fish carcasses in August during daily surveys of spawning areas, including C Creek, nearby A Creek and shoreline areas near the two stream outlets. We recovered 40 temperature loggers from salmon carcasses.

### Monitoring water temperature

To characterize the range of water temperatures available to sockeye salmon during peak spawning, we placed iButton temperature data loggers in the lake hypolimnion (30 m), the lake epilimnion (1 m), the river draining the lake and three tributary streams (A, C and Little Togiak creeks). Epilimnion temperatures loggers were placed at three sites spread across the lake (one of the loggers did not begin recording until 27 July, the onset of fish temperature monitoring). Water temperatures were recorded every 20 min.

### Data analysis

We truncated the time-series data for fish temperatures to include only the period spanning 1 h after tagging until the fish entered a spawning tributary stream (either A or C Creek). A and C Creeks are groundwater dominated and exhibit a relatively stable water temperature of 3.6 ± 0.7°C (mean ± SD from iButtons), which is much cooler than the lake surface temperature near the stream outlets. We identified stream entry of individual fish as occurring when the recorded temperatures from sockeye iButtons matched that of the stream.

To evaluate whether sockeye salmon thermoregulate, we constructed a hierarchical model relating the lake epilimnion water temperatures (predictor, *water*_*i*,*t*_) to the water temperatures occupied by fish (response, *fish*_*i*,*t*_) based on the thermal regime collected from iButtons attached to individual fish. We allowed the effect of water temperature to vary hierarchically, so that ${\mathrm{fish}}_{i,t}=B_{0,i}+B_{1,i}{\mathrm{water}}_{i,t}+\varepsilon_{i,t}$, where $B_{0,i}$ and $B_{1,i}$ represent the intercept and slopes for individual *i*, and are modelled as $B_{0,i}\sim \mathrm{Normal}\left(u_{B0},\sigma_{B0}\right)$ and $B_{1,i}\sim \mathrm{Normal}\left(u_{B1},\sigma_{B1}\right)$, and $\varepsilon_{i,t}$ represents residual variation assumed to be normally distributed. Analysing the data in a hierarchical framework allowed us to estimate the overall thermoregulatory response, as well as variation among individual fish. This approach also allowed us to calculate derived metrics, such as the water temperatures for individual fish that corresponded to equilibrium (epilimnion water temperature equalling fish temperature). In our analysis, we used water temperature data from the epilimnion site closest to where we tagged the fish (~30 m from tagging site).

### Comparison of 2013 temperatures with historical records

We evaluated the 2013 water and air temperatures at our study site in a historical context to determine whether the conditions during our study were indeed anomalously warm. We considered temperatures during the seasonal window of 1 July–15 August, which represents the staging period between freshwater entry and the peak of spawning in A and C Creeks (Lisi *et al*., 2013). We characterized inter-annual variation in epilimnion water temperature from limnology surveys dating back to 1958, calculating the maximal observed temperature during each year's staging period (*n* = 21 because of missing years). Although these data allowed us roughly to characterize annual variation in thermal maxima, they lacked the temporal resolution needed to capture ephemeral phenomena, such as heat waves. We complimented these temporally coarse water temperature records with daily air temperature records from the King Salmon weather station, which is ~130 km from our study site and exhibits similar air temperatures. A weather station in our focal system (at Lake Aleknagik, ~35 km from Little Togiak Lake) recording air temperature during 2013 showed strong correlation with the King Salmon station (maximual air temperatures: *r*^2^ = 0.93, *P* < 0.001, *n* = 214 days). To evaluate whether the heat wave of 2013 represented a weather anomaly, we calculated a 3 day running mean for each staging period during the years 1942–2014 and identified the highest mean (i.e. warmest heat wave) for each year.

### Estimation of the energetic implications of thermoregulation

In order to assess the energetic consequences of thermal habitat selection, we calculated the metabolic cost of active metabolism for a 2.5 kg sockeye salmon (a typical sized individual in our study system) using the equations from the Wisconsin Bioenergetics model (Hanson, 1997) with the physiological parameter set for sockeye salmon (Beauchamp *et al*., 1989). We simulated accrued energetic costs from 1 July to 15 August (the approximate staging period) based on the following four scenarios of thermal habitat selection: (i) a fish that held in the epilimnion at all times; (ii) a fish that held in the epilimnion when the water temperature was <12°C, but exploited cooler water in the metalimnion or tributary plumes such that it never experienced temperatures >12°C; (iii) a fish that always held in 12°C water; and (iv) a fish that always held in the hypolimnion. We translated accrued metabolic costs into percentage energy loss by assuming an initial fish energy density of 6000 J g^−1^ (Hendry and Berg, 1999) and comparing the accrued costs with the total energy of the 2.5 kg fish at the beginning of the simulation.

## Results

### Water and air temperature in 2013 compared with historical records

**Figure 2:**
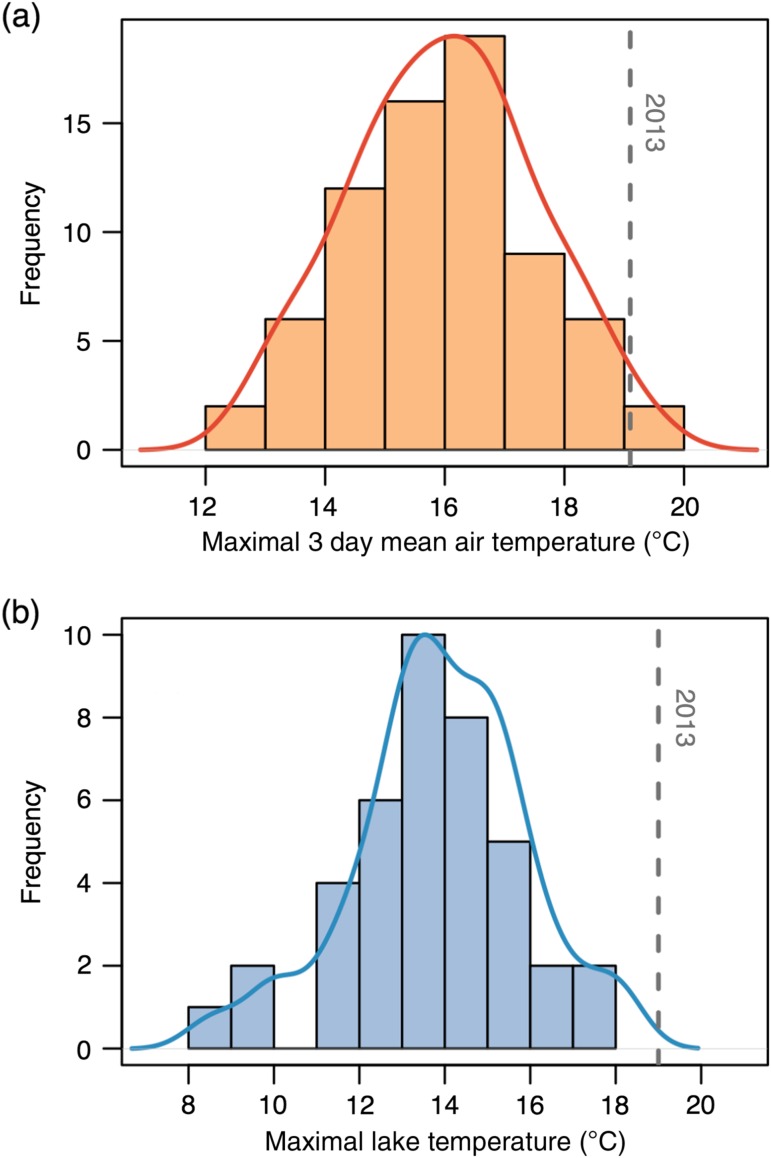
Historical records of air temperature and water temperature during the period when sockeye salmon stage in Little Togiak Lake, Alaska (1 July–15 August). (**a**) Histogram showing the maximal 3 day running average of air temperature for the years 1942–2014. Dashed vertical line indicates the 3 day average recorded during the heat wave of 2013. (**b**) Histogram showing the maximal epilimnion water temperatures from limnology surveys of Little Togiak Lake dating back to 1958 (*n* = 21 owing to years without sampling). Sampling occurred approximately every 2 weeks during each year. Dashed vertical line indicates the maximal epilimnion temperature recorded during continuous monitoring in 2013 (averaged across three sites). In both histograms, the superimposed line shows a kernel density estimate.

The maximal 3 day average air temperature during the 1 July–15 August staging period ranged from 12.7 to 19.4°C during the years 1942–2014. Our study year exhibited a maximal 3 day average of 19.1°C, representing the second warmest heat wave on record (Fig. 2). During the 2013 heat wave, the maximal water temperature recorded in the epilimnion ranged from 18.4 to 19.9°C among our three monitoring sites. In the 21 years of limnology records dating back to 1958, the average maximal water temperature recorded was 14.2 ± 2.0°C. (mean ± SD for temperatures taken between 1 July and 15 August); the highest individual water temperature recorded during limnology surveys was 18°C in 2005 (Fig. 2). These data suggest that the heat wave recorded during 2013 was indeed a unique event that exposed fish to novel conditions of heat stress.

### Water temperatures available to sockeye salmon prior to spawning

**Figure 3:**
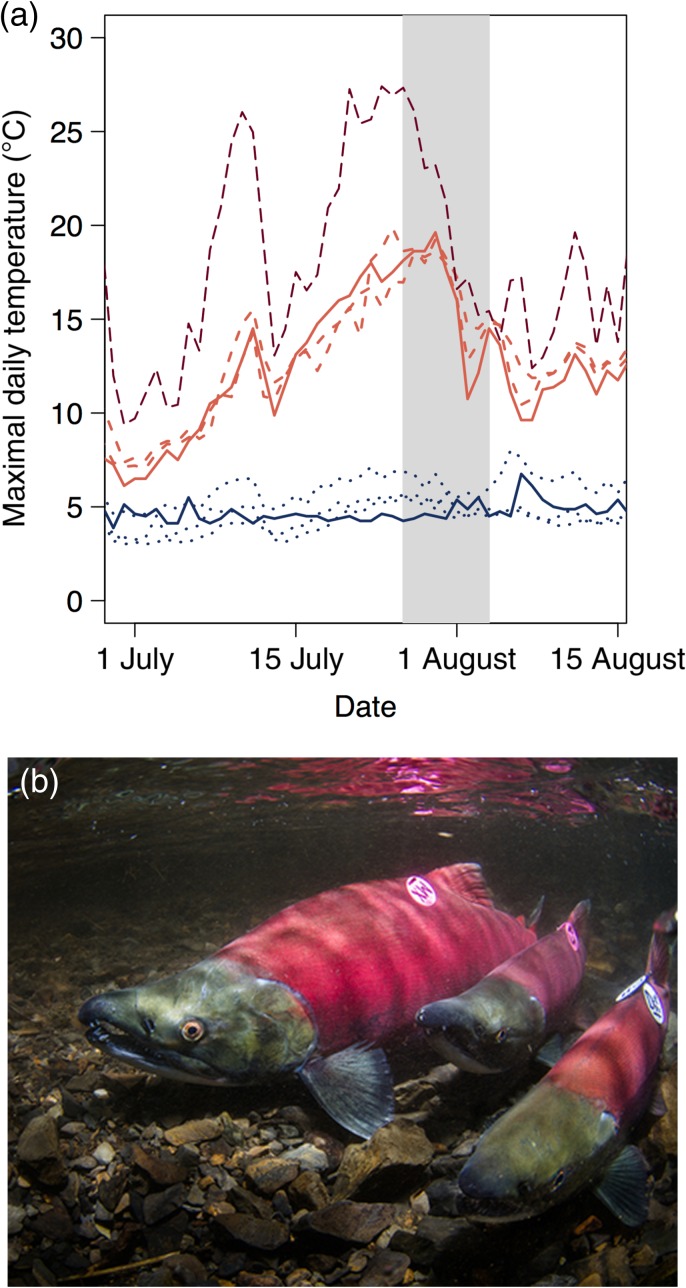
(**a**) Time series of water temperature for different habitats in Little Togiak Lake, including tributary streams (blue short dashes; A, C and Little Togiak Creeks). the hypolimnion (blue continuous line). the epilimnion (light red long dashes) and the lake outlet (red continuous line). Dark red dashed line indicates the air temperature at nearby Lake Aleknagik. Shaded box indicates the temporal extent over which fish temperatures were monitored in Little Togiak Lake. We tagged all fish on 27 July, and the last fish entered C Creek on 4 August. (**b**) Image of our study fish (sockeye salmon) after they had left Little Togiak Lake to spawn in C Creek.

The thermally heterogeneous habitat of Little Togiak Lake and its tributaries provided a wide range of temperature options for sockeye salmon (Fig. 3). At the cold end of the spectrum, the hypolimnium of the lake was ~4–5°C, and the groundwater-dominated tributaries A and C Creeks ranged from ~3 to 6°C. The warmest temperatures occurred in the lake epilimnion and lake outlet, where the average daily maximal temperature was 15.0°C, and the highest temperature recorded across all sites was 19.9°C (Fig. 3).

### Water temperatures selected by tagged sockeye salmon

**Figure 4:**
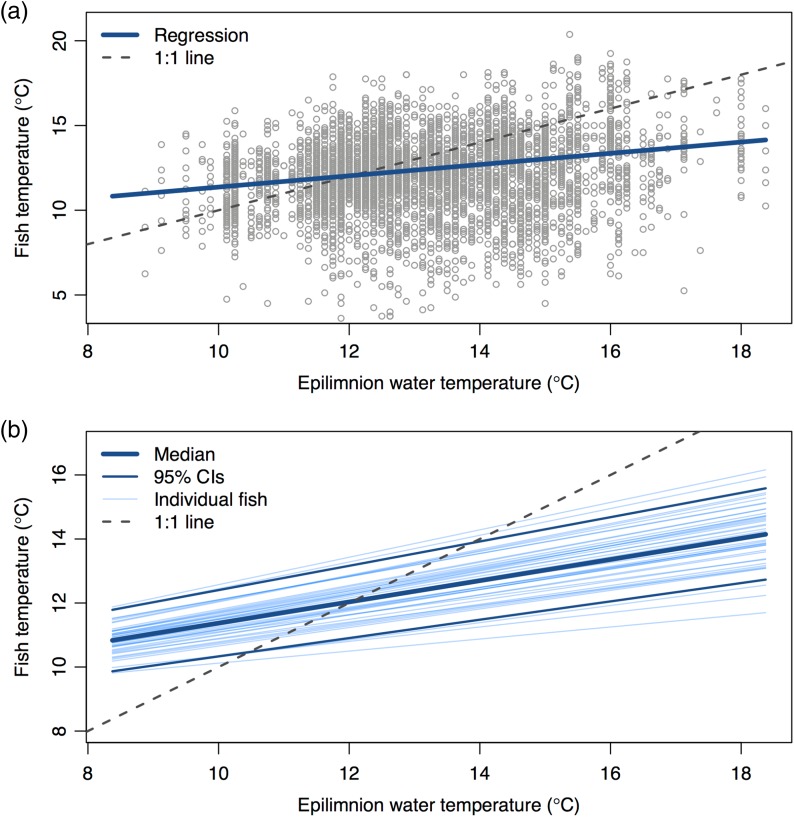
(**a**) The relationship between epilimnion water temperature and the water temperatures recorded by loggers attached to adult sockeye salmon holding in Little Togiak Lake. Bold blue line indicates linear regression; dashed line indicates a 1:1 relationship depicting thermal conformity. (**b**) Variation among individuals in the estimated slope parameter for the hierarchical Bayesian regression model in panel (a) (light blue lines). Dashed line indicates 1:1; dark blue lines represent median and 95% confidence intervals (CIs).

**Figure 5:**
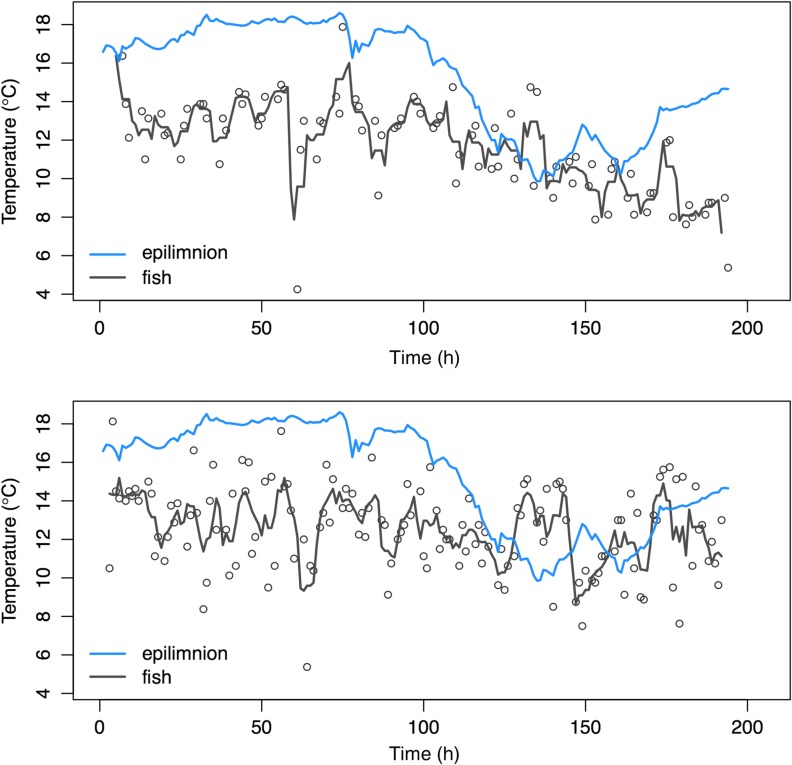
The thermal histories of two sockeye salmon, shown as examples. Each panel shows the epilimnion water temperature (blue line) and the temperatures recorded every 20 min by loggers attached externally to sockeye salmon (black open circles). The black line shows a running mean with a window of ±2 observations. The time series start on 27 July.

We recovered 40 of the 95 temperature loggers that were deployed. The tags varied in their duration of monitoring, because some fish entered the stream earlier than others (123 average observations per fish, range = 25–473 observations per fish, logging interval = 20 min). Sockeye salmon sampled a wide range of temperatures (~4–20°C; Fig. 4) but exhibited a clear pattern of thermoregulation, generally avoiding temperatures higher than ~15°C (Figs 4a and 5). Our hierarchical model included individual random effects, interpreted as inter-individual variation in the thermoregulatory response of fish and their environment. Compared with a simpler null model without random effects, the inclusion of random effects was supported using model selection (Δdeviance information criterion = 47; Spiegelhalter *et al*., 2002). The posterior distribution of the global slope ($u_{B0}$) was positive (mean = 0.34, 95% confidence interval 0.27–0.43), and the posterior means of the slopes for individual fish ranged from 0.071 to 0.51 (Fig. 4b). We also used each Markov chain Monte Carlo draw to calculate the equilibrium temperature where the difference between fish and water was zero. The global mean equilibrium was 12.1°C (95% confidence interval 10.9–13.2°C), and the means of individual fish ranged from 10.0 to 14.9°C (confidence intervals across all fish ranged from 8.6 to 17.0°C; Fig. 4b).

### Energetic implications of alternative thermoregulatory behaviour

For a 2.5 kg sockeye salmon, the daily cost of active metabolism was estimated at 77, 172 and 323 kJ at 4, 12 and 18°C, respectively. These three temperatures approximately represent the contrasting thermal conditions among the hypolimnion or spring-fed tributaries (4°C), habitats selected by sockeye salmon (12°C) and the epilimnion on a warm day (18°C). In our 45 day simulations based on observed temperatures, a fish holding in the epilimnion used ~18% of its total energy, whereas a fish holding in the hypolimnion used only 7%, and one holding at a constant 12°C used 16%. A fish holding in the epilimnion but thermoregulating to avoid temperatures in excess of 12°C used 14% of its total energy.

## Discussion

We documented behavioural thermoregulation in a high-latitude population of sockeye salmon experiencing novel exposure to stressful thermal conditions. During a period when temperatures in the lake epilimnion reached daily maxima of ~15–20°C, sockeye salmon moved to the cooler waters at the edges of tributary plumes or the lake metalimnion, selecting for water temperatures of ~12°C (Fig. 4). The observed pattern of thermoregulation, if expressed across the 45 day staging period, would have reduced metabolic costs by ~640 kJ, which represents ~4 days of energetic costs. The vast majority of simulated energetic savings (76%) occurred during the 8 day period when epilimnion temperatures exceeded 15°C (i.e. the heat wave). Thermoregulation may also reduce susceptibility to disease (Bradford *et al*., 2010), but the quantitative nature of this relationship remains uncertain (Lafferty, 2009). Cool-seeking behavioural thermoregulation has been demonstrated in the core or southern portion of salmonid distributions, but we provide the first evidence of this coping tactic in a population at the northern extent of its range, where suboptimally warm temperatures are rare (i.e. annual thermal maximum averages <15°C).

Given that we tagged fish externally, the temperature loggers recorded instantaneous readings of the water temperatures experienced by fish. The real body temperatures of fish integrate across time and are likely to reflect a smoothed version of our observed data (Pépino *et al*., 2015) similar to the temperature values predicted by the regression model (Fig. 4). The high level of variation in the fish temperature data (Figs 4a and 5) suggests that fish were frequently moving across thermoclines, spatially integrating across fine-scale thermal variation. Based on our observations, most of the fish we tagged resided at the edge of the C Creek plume, at the interface between cold stream water and the warm lake epilimnion. The stream plume is quite small (~3 m wide) and exhibits fine-scale thermal heterogeneity that shifts over time as a result of wave action and other factors. We hypothesize that fish are constantly repositioning to tune their body temperature (Fig. 5). Prior examples of thermoregulation in fishes have mostly occurred in environments where the spatial scale of thermal habitat patches is large compared with that of the fish (e.g. the vertical zones of lakes or segments of a stream network).

The time period over which we observed sockeye salmon was relatively short because of the opportunistic nature of our study and because the fish we tagged ended up comprising the early portion of the C Creek salmon run. Testing for behavioural thermoregulation over a short time period can be problematic because fish are less likely to be exposed to a broad range of temperatures and there is less statistical power to detect behavioural responses. Fortunately, our opportunistic monitoring captured a range of temperatures spanning from physiologically optimal (~9–15°C) to conditions known to cause declines in physiological performance and fitness (>15°C; Brett 1971; Eliason *et al*., 2011). The behavioural response of fish was strong enough to reveal a clear signal of thermoregulation (Fig. 4).

Sockeye salmon in Little Togiak Lake behaviourally thermoregulated to a preferred temperature of ~12°C, an optimal temperature for maturation based on review of empirical studies (McCullough *et al*., 2001). When temperatures in the epilimnion fell below 12°C, we observed warm-seeking thermoregulatory behaviour. This is likely to reflect fish residing in microstratified water shallower than our 1-m-deep temperature loggers in the epilimnion (Coloso *et al*., 2011). Similar to the results of Newell and Quinn (2005), sockeye salmon in our system selected intermediate temperatures compared with what was available, avoiding colder temperatures in the hypolimnion or tributary plumes even though they could have reduced metabolic costs by ~50%. This suggests that intermediate temperatures confer a physiological advantage, perhaps needed to complete sexual maturation prior to initiation of spawning (Newell and Quinn, 2005; Roscoe *et al*., 2010). We detected individual variation in thermoregulation, but the 95% confidence interval for the preferred temperature exhibited a fairly narrow range, from 10.9 to 13.2°C (Fig. 4b). Roscoe *et al*. (2010) documented substantial individual variation in thermoregulation, such that females with low energy reserves selected cooler temperatures. The fact that we did not observe stronger individual variation in thermoregulatory tactics may be because there is limited variation in the energetic condition of returning adult salmon. Hendry and Berg (1999) found that sockeye salmon in the Wood River depleted the majority of their energy stores during the period between freshwater entry and the onset of spawning, but that fish exhibited minimal inter-individual variation in fat stores at either the beginning or the end of this time period. It is possible that low-condition individuals stay in the ocean for an additional year rather than returning to spawn.

Groot and Margolis (1991) consider the primary northern distribution of sockeye salmon to end at the Kuskokwim River, which enters the Bering Sea at roughly the same latitude as our study site, 60°. Coastal watersheds (or adjacent seas) at these latitudes rarely exhibit water temperatures beyond the thermal optimum of Pacific salmon, yet climate models suggest that these northern ecosystems will experience particularly high levels of warming in the decades to come (Schneider and Hook, 2010; Abdul-Aziz *et al*., 2011). Our study provides some cautious optimism about the ability of sockeye salmon to persist in a warmer future in this watershed, assuming that the main migratory rivers do not warm sufficiently to impede migration (Hyatt *et al*., 2003). In an unusually warm year that provided a glimpse of conditions to come, sockeye salmon thermoregulated behaviourally, holding in cool habitats that were as much as ~7°C cooler than that of the lake epilimnion. This behaviour decreased metabolic costs and prevented exposure to temperatures known to cause physiological stress (Bradford *et al*., 2010). Although we did not directly measure survival, a concurrent study marking sockeye salmon in Little Togiak Lake with visual ID tags subsequently resighted 94% of individuals on the spawning grounds of A and C Creeks (R. Hilborn, University of Washington, unpublished data).

The thermal heterogeneity of our intact focal watershed, The Wood River System, supports a diversity of behavioural tactics and illustrates the potential significance of restoring heterogeneity in degraded ecosystems elsewhere. Recent studies from the Wood River system found juvenile coho salmon in tributary streams exhibiting warm-seeking thermoregulation during the same time period that the adult salmon in our study were seeking cooler water (Armstrong and Schindler, 2013; Armstrong *et al*., 2013). While these fishes exploit thermal heterogeneity for direct physiological benefits, grizzly bears and other salmon-eating consumers exploit indirect ecological benefits, tracking temperature-induced phenological variation among salmon populations to prolong high-quality foraging opportunities (Ruff *et al*., 2011; Schindler *et al*., 2013). Watershed restoration efforts, such as dechannelization, beaver reintroduction and floodplain rehabilitation, are increasing thermal heterogeneity in degraded ecosystems. We encourage behavioural and physiological studies that evaluate how these efforts facilitate the expression of thermoregulation and other tactics by which animals cope with environmental variation and climate change.
